# Influence of the cystic fibrosis transmembrane conductance regulator on expression of lipid metabolism-related genes in dendritic cells

**DOI:** 10.1186/1465-9921-10-26

**Published:** 2009-04-03

**Authors:** Yaqin Xu, Christine Tertilt, Anja Krause, Luis EN Quadri, Ronald G Crystal, Stefan Worgall

**Affiliations:** 1 Department of Pediatrics, Weill Cornell Medical College, New York, USA; 2 Department of Genetic Medicine, Weill Cornell Medical College, New York, USA; 3 Department of Microbiology and Immunology, Weill Cornell Medical College, New York, USA; 4 Department of Immunology, Johannes Gutenberg University, Mainz, Germany

## Abstract

**Background:**

Cystic fibrosis (CF) is caused by mutations in the cystic fibrosis transmembrane conductance regulator (CFTR) gene. Infections of the respiratory tract are a hallmark in CF. The host immune responses in CF are not adequate to eradicate pathogens, such as *P. aeruginosa*. Dendritic cells (DC) are crucial in initiation and regulation of immune responses. Changes in DC function could contribute to abnormal immune responses on multiple levels. The role of DC in CF lung disease remains unknown.

**Methods:**

This study investigated the expression of CFTR gene in bone marrow-derived DC. We compared the differentiation and maturation profile of DC from CF and wild type (WT) mice. We analyzed the gene expression levels in DC from naive CF and WT mice or following *P. aeruginosa *infection.

**Results:**

CFTR is expressed in DC with lower level compared to lung tissue. DC from CF mice showed a delayed in the early phase of differentiation. Gene expression analysis in DC generated from naive CF and WT mice revealed decreased expression of Caveolin-1 (Cav1), a membrane lipid raft protein, in the CF DC compared to WT DC. Consistently, protein and activity levels of the sterol regulatory element binding protein (SREBP), a negative regulator of Cav1 expression, were increased in CF DC. Following exposure to *P. aeruginosa*, expression of 3β-hydroxysterol-Δ7 reductase (Dhcr7) and stearoyl-CoA desaturase 2 (Scd2), two enzymes involved in the lipid metabolism that are also regulated by SREBP, was less decreased in the CF DC compared to WT DC.

**Conclusion:**

These results suggest that CFTR dysfunction in DC affects factors involved in membrane structure and lipid-metabolism, which may contribute to the abnormal inflammatory and immune response characteristic of CF.

## Introduction

Cystic fibrosis (CF) is caused by mutations in the cystic fibrosis transmembrane conductance regulator (CFTR) gene, a member of the ATP-binding cassette (ABC) protein family that functions as a cAMP-dependent chloride channel [1-4]. ABC transport proteins play important roles in a variety of tissues including lung, liver, pancreas and the immune system[2]. Although CF is primarily thought to be a disease of abnormal salt and fluid transport caused by the defective chloride channel function of the CFTR protein, dominant additional features of defective CFTR include an exaggerated inflammatory response and susceptibility to microbial colonization in the lung, particularly with *P. aeruginosa *[5-7]. The exact mechanism for this is not completely understood. Overall in CF, host immune responses do not seem to be adequate to eradicate *P. aeruginosa *from the respiratory tract. Attention in this regard has been primarily focused on the role of CFTR in epithelial cells [8-10]. However, functional expression of CFTR has been demonstrated in a variety of non-epithelial cells, including lymphocytes, neutrophils, monocytes, macrophages and endothelial cells [11-15]. The widespread distribution of CFTR expression in non-epithelial cells and cells of the immune system implies a variety of functions, including a possible regulatory role in the secretion of cytokines and antibodies by lymphocytes and regulation of lipopolysaccharide (LPS) and interferon-γ-induced macrophage activation[15,16]. In murine alveolar macrophages CFTR-expression is related to lysosomal acidification and intracellular killing of *P. aeruginosa *[15], and macrophages directly contribute to the exaggerated inflammatory response in CFTR knockout mice [17]. The interaction of the CF-specific infectious organisms with cells of the host immune system are likely important in determining the extent of the inflammatory responses and the subsequent clearance of the bacteria from the airways [6,18,19].

Abnormalities in the lipid metabolism have been described in CF patients [20], and have been suggested to be related to the inflammatory responses in CF [19-21]. Deficiency of essential fatty acids is thought to be primarily a result of defective intestinal fat absorption secondary to a deficiency of pancreatic lipase due to obstruction of the pancreatic ducts [20]. It has furthermore been suggested that mutant CFTR plays a role in cellular essential fatty acid utilization [20,22]. The misassembled deltaF508 CFTR leads to altered cellular lipid trafficking in the distal secretory pathway [21]. Localization of CFTR to lipid rafts, cellular lipid membrane domains that are enriched cholesterol and sphingolipids, has been described following infection with *P. aeruginosa*, and has been linked to inflammatory signaling and apoptosis [23-25].

The present study analyzed dendritic cells (DC) derived from CF and WT mice. DC are the most potent antigen presenting cells and are crucial in the initiation and regulation of immune responses [26-29]. Changes in DC function could contribute to abnormal immune responses on multiple levels, such as antigen processing and presentation, expression of costimulatory molecules, and production of cytokines [26-29]. The DC from CF mice were delayed in their differentiation compared to the WT mice, but were able to reach fully maturation after 8 days. Interestingly, of the relatively few genes found to be down-regulated comparing CF and WT DC in gene expression studies, was Caveolin-1 (Cav1), a lipid raft membrane protein related to the cellular lipid metabolism. The protein expression and activity of the sterol regulatory element binding protein (SREBP), a negative regulator of Cav1 expression [30-32], was increased in CF DC compared to WT DC. Among the genes showing expression change comparing WT and CF DC upon *P. aeruginosa *infection, were 3β-hydroxysterol-Δ7 reductase (Dhcr7) and stearoyl-CoA desaturase 2 (Scd2), two enzymes involved in the lipid metabolism that are also regulated by SREBP [33-37]. This study provides insight into CFTR-dependant gene expression abnormalities related to the cellular lipid homeostasis in a non-epithelial cell type.

## Materials and methods

### Mice

Congenic C57BL/6J heterozygous breeding pairs (*Cftr*^tm1UNC^) were maintained on regular mouse chow and continuously bred. To maintain congenic status and prevent genetic drift, each new generation of mice was bred to WT C57BL/6J mice, obtained from Jackson Laboratories (Bar Harbor, ME). Male and female WT (*cftr+/+*) animals were used in alternate breeding. Offspring were genotyped at 14 days of age by PCR analysis of tail-clip DNA. To minimize bowel obstruction and optimize long-term viability, 21- to 23-day-old CF mice (C57BL/6J *Cftr *^tm1UNC^/*Cftr*^tm1UNC^) and their *cftr+/+ *littermates were fed a liquid diet (Water and Peptamen, Nestle Nutrition) provided ad libitum. All procedures were approved by the Institutional Animal Care and Use Committee of Weill Cornell Medical College.

### Bone marrow-derived dendritic cells (DC)

DC, generated from mouse bone marrow precursors from the three pair of CF mice and their WT littermates with age 5 to 6 wk old, were cultured in RPMI 1640 medium supplemented with 10% fetal bovine serum (FBS), penicillin (100 U/ml), streptomycin (100 μg/ml) (Invitrogen Corporation, CA), recombinant murine granulocyte-macrophage colony-stimulating factor (GM-CSF, 10 ng/ml; R&D System, MN) and recombinant murine interleukin-4 (IL-4, 2 ng/ml; R&D System), for 8 days as previously described [38]. DC represent the mature DC population after differentiation for 8 days.

Aliquots of DC were harvested, and differentiation and maturation profiles were analyzed on day 0, 2, 4, 6 and 8 for expression of CD11c and CD40, CD40L, CD80, CD86, ICAM, MHCI or MHCII (BD Pharmingen, CA) by flow cytometry (FACS Calibur, BD, CA). On day 8 more than 85% of the cells were mature DC. The assays have been carried out at least three times.

### DC Infection with *P. aeruginosa*

The *P. aeruginosa *strain used was the laboratory strain PAK (kindly provided by A. Prince, Columbia University, NY). Bacteria were grown from frozen stocks in tryptic soy broth (Difco, MI) at 37°C to mid-log phase, washed three times with phosphate buffered saline (PBS) pH 7.4 (Invitrogen Corporation), and resuspended in the infection media at the desired concentration as determined by spectrophotometry. The DC were incubated for 4 h with 10 CFU of PAK per cell in RPMI 1640 supplemented with 25 mM Hepes (Biosource International, MD) and then harvested for RNA and protein extraction.

### CFTR Expression in DC

RNA was extracted from lung and DC from three WT mice using TRIzol (Invitrogen Corporation). Following reverse transcription of 2 μg RNA, CFTR mRNA was amplified by real-time RT-PCR using a CFTR-specific probe (Mm00445197_m1, Applied Biosystems, CA). The CFTR mRNA levels were quantified using the ΔΔCt method (Ambion, Instruction Manual) and normalized relative to GAPDH (Applied Biosystems). The PCR reactions for CFTR and GAPDH were optimized to have equal amplification efficiency.

CFTR protein levels were determined by Western analysis. Total cellular fractions were isolated from mouse lung and DC. Following determination of protein concentration (Micro BCA™ Protein Assay Kit; PIERCE, IL), 30 μg protein was separated by electrophoresis on NuPAGE@Novex 4–12% Bis-Tris Gel (Invitrogen Corporation), transferred to a polyvinylidene difluoride (PVDF) membrane (Bio-Rad Laboratories, CA) and incubated with a rabbit anti-CFTR antibody (1:200, Santa Cruz Biotech Inc., CA). Horseradish Peroxidase-conjugated goat anti-rabbit IgG secondary antibody (1: 3000, Bio-Rad Laboratories) and Amersham ECL Plus Western Blotting System (GE Healthcare Bio-Sciences Corp., NJ) were used for detection. Following scanning, the membranes were stripped with stripping buffer (100 mM 2-Mercaptoethanol, 2% SDS, 62.5 mM Tris-HCl, pH 6.7) and re-blotted using a mouse anti-GAPDH antibody (1:5000, Abcam Inc. MA). CFTR levels relative to GAPDH levels were quantified using Image J software [39]. The assays have been carried out at least three times.

### Preparation of RNA for Microarray Analysis and Processing of Microarrays

All analyses were carried out with the Affymetrix MG-U74Av2 GeneChip using the protocols from Affymetrix (Santa Clara, CA). DC were purified from six mice with age 5 to 6 wk old. Total RNA was extracted from the DC using TRIzol followed by RNeasy (Qiagen, CA) to remove residual DNA. First strand cDNA was synthesized using the T7-(dT)_24 _primer (sequence 5'-GGC CAG TGA ATT GTA ATA CGA CTC ACT ATA GGG AGG CGG-(dT)_24_-3', HPLC purified from Oligos Etc., OR) and converted to double stranded cDNA using Superscript Choice system (Life Technologies). Double stranded cDNA was purified by phenol chloroform extraction and precipitation and the size distribution assessed by agarose gel electrophoresis. This material was then used for synthesis of the biotinylated RNA transcript using the BioArray HighYield reagents (Enzo), purified by the RNeasy kit (Qiagen) and fragmented immediately before use. The labeled cRNA was first hybridized to the test chip and then, when satisfactory, to the MG-U74Av2 GeneChip for 16 h. The GeneChips were processed in the fluidics station under the control of the Microarray Suite software (Affymetrix) to receive the appropriate reagents and washed for detection of hybridized biotinylated cRNA and then manually transferred to the scanner for data acquisition.

### Microarray Data Analysis

The image data on each individual microarray chip was scaled to arbitrary target intensity, using the Microarray Suite version 5.0 (MAS 5.0). The raw data was normalized using the GeneSpring GX 7.3.1 software (Agilent Technologies, CA) by setting measurements <0.01 to 0.01, followed by per-chip normalization to the 50^th ^percentile of the measurements for the array, and per-gene by normalizing to the median measurement for the gene across all the arrays in the data set. Data from probe sets representing genes that failed the Affymetrix detection criterion (labeled "Absent" or "A", or "Marginal" or "M") in over 90% of microarrays were eliminated from further assessment. All further analyses were carried out on the remaining 6,474 genes selected using this criterion.

Genes with significantly different expression levels in WT and CF DC with and without infection with *P. aeruginosa *were annotated using the NetAffx Analysis Center http://www.affymetrix.com to retrieve the Gene Ontology (GO) annotations from the National Center for Biotechnology (NCBI) databases. For probe sets with no GO annotations, other public databases [Mouse Protein Reference Database, Kyoto Encyclopedia of Genes and Genomes (KEGG), PubMed] were searched. These genes were grouped into 8 subcategories: (1) immunity; (2) metabolism/enzyme; (3) signal transduction/growth control; (4) protein biosynthesis/cell adhesion; (5) cell cycle; (6) transcription; (7) transport and (8) not classified genes.

Comparisons of the gene profile difference between WT and CF naive DC, and DC following infection with *P. aeruginosa *were carried out using the normalized data using the Welch's approximated t-test with Benjamini-Hochberg multiple testing correction. This analysis was done on the 6,474 genes that passed the Affymetrix detection criterion (labeled " Present") in over 10% of the samples, and genes were assumed to be significantly up-regulated or down-regulated if the calculated p-value was < 0.05 and the fold change was greater than 1.5 up or down. All data was deposited at the Gene Expression Omnibus site http://www.ncbi.nlm.nih.gov/geo/, a high-throughput gene expression/molecular abundance data repository curated by the National Center for Bioinformatics site. The accession number for the MG-U74Av2 data set is GSE9488.

### Confirmation of Microarray Data by Real-time RT-PCR

Messenger RNA levels of CFTR, Cav1, Dhcr7 and Scd2 were confirmed using real-time quantitative RT-PCR, using gene specific probes (CFTR: Mm00483057_m1, Cav1: Mm00483057_m1, Dhcr7: Mm00514571_m1, and Scd2: Mm01208542_m1, Applied Biosystems) on independent samples. RNA levels were quantified by real-time quantitative RT-PCR with fluorescent TaqMan chemistry using the ΔΔCt method, as described above and normalized to GAPDH mRNA. The assays have been carried out at least three times.

To reconfirm the genotype of cDNA samples from CF and WT DC, the primers mCF19 (exon10-11, 5'-TGGATCAGGAAAGACATCACTC-3') and mCF20 (exon 14, 5'-TTGGCCATCAATTTACAAACA-3') were used for PCR amplification. The reaction was amplified for 35 cycles at 94°C/30s (denature), 58°C/30s (annealing), and 72°C/45s (extension). The GAPDH gene primers were used as the PCR endogenous control (Applied Biosystem, CA). The reaction was amplified for 35 cycles at 94°C/30s (denature), 58°C/30s (annealing), and 72°C/30s (extension). PCR products were analyzed on 2% Agarose-LE gel (Applied Biosystems), stained with ethidium bromide and visualized under UV light.

### Cav1 and SREBP Protein Expression

Total cellular fractions were isolated from naive DC and DC infected with *P. aeruginosa *from three pair of CF and WT mice. Cav1 and SREBP were determined by Western analysis using a rabbit anti-Cav1 antibody (1:200, Santa Cruz Biotech, Inc.) and a rabbit anti-SREBP antibody (kindly provided by T. Worgall, Columbia University, NY), detailed procedures as described above. Cav1 and SREBP levels were normalized to GAPDH (mouse anti-GAPDH, 1:5000, Abcam Inc). Cav1 and SREBP protein levels relative to GAPDH levels were quantified using Image J software [39]. The assays have been carried out at least three times.

### SRE Activity in CF DC

The transcriptional activity of SRE in CF DC was assessed using an adenovirus vector expressing the SRE-promoter of HMG-CoA synthase linked to a luciferase reporter gene and β-galactosidase gene (AdZ-SRE-luc) (kindly provided by T. Worgall, Columbia University, NY) by luciferase assay. The CF and WT DC were infected with AdZ-SRE-Luc for 48 h, and then infected with *P. aeruginosa *for 4 h. Luciferase and β-galactosidase activities were analyzed in the cell lysates by luminometric luciferase and β-galactosidase assays (both, Stratagene, CA). Luci-ferase activity (RLU) was quantified by luminometer (Pharmingen) and β-galactosidase levels by microplate luminometer (Bio-Rad Laboratories). The data is expressed as luciferase activity (RLU) normalized to β-galactosidase activity.

## Results

### CFTR Expression in DC from WT Mice

**Figure 1:**
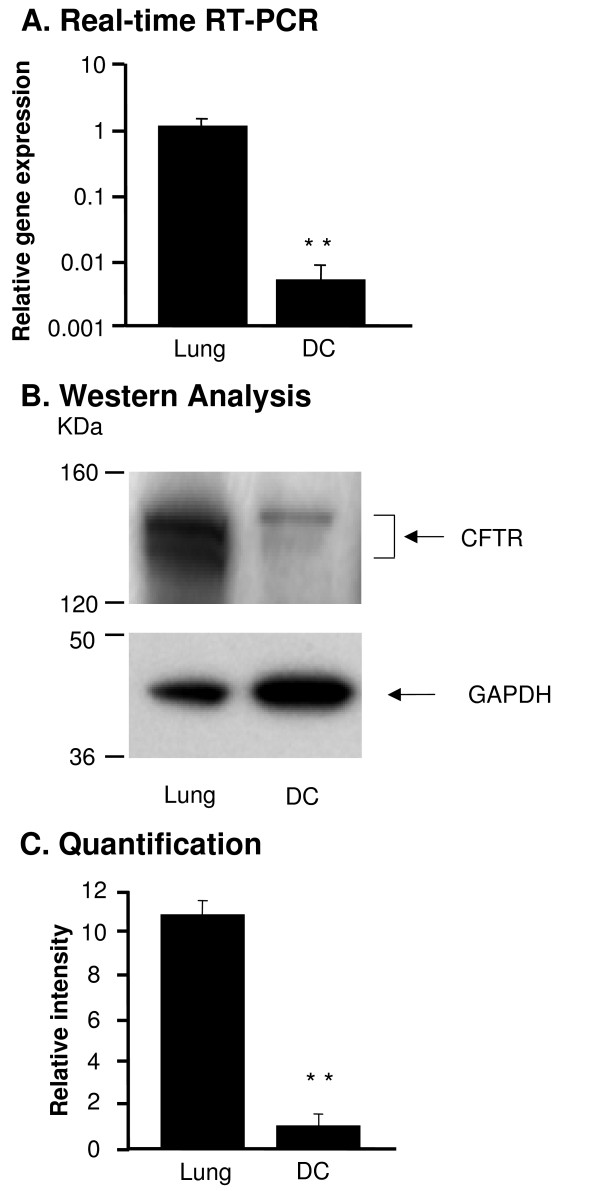
**CFTR expression in bone marrow derived dendritic cells (DC)**. RNA and protein were extracted from wild type (WT) mouse lung and DC. CFTR expression was measured by real-time RT-PCR and Western analysis. A. Real-time RT-PCR. WT mouse lung tissue was used as a positive control and calibration. The *y*-axis represents CFTR cDNA transcription level in terms of relative quantity value (RQ). B. Western analysis of CFTR protein in DC compared to the WT lung tissue. C. Quantification of CFTR protein by image intensity analysis. Images were scanned and analyzed by software Image J normalized to GAPDH loading control. Shown is the mean ± SEM of three pairs of independent samples. **denotes p < 0.01.

First we evaluated the level of CFTR expression in DC compared to lung tissue known for high expression of CFTR. CFTR mRNA was detected in DC and whole lung by real-time RT-PCR (Figure 1A). The CFTR mRNA levels were 212-fold lower in the DC compared to the whole lung (p < 0.01). Likewise, CFTR protein was detected by Western analysis (Figure 1B); the expression level in DC was 11-fold lower compared to lung (p < 0.01, Figure 1C).

### Gene Expression Difference in DC from WT and CF Mice

**Figure 2:**
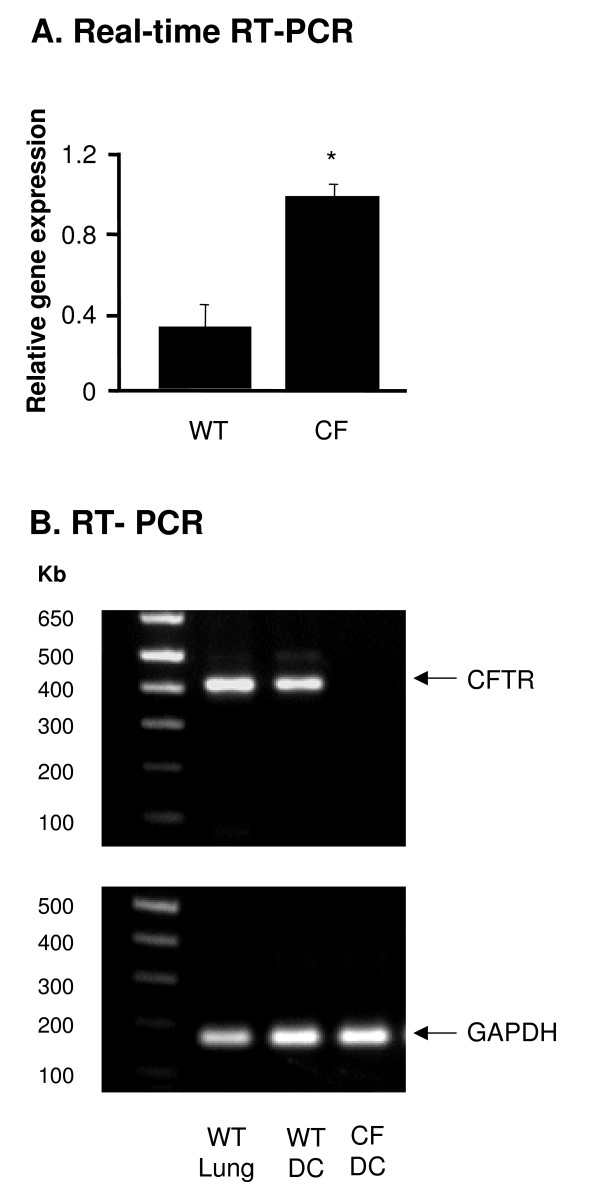
**CFTR expression in DC from *Cftr*^tm1UNC ^mice**. RNA was extracted from WT and *Cftr*^tm1UNC ^(CF) DC. CFTR expression was measured by real-time RT-PCR and reverse-transcription PCR. A. Real-time RT-PCR. Relative expression levels in the samples were calculated using the ΔΔCt method, using GAPDH as internal normalization control. The *y*-axis represents CFTR cDNA transcription level in terms of relative quantity value (RQ). B. Reverse-transcription PCR of CFTR in DC from WT and CF mice. Lung from WT mice were used as positive control. Primers were designed to detect WT CFTR cDNA but not mutant CFTR. GAPDH was used as endogenous PCR control. Shown is the mean ± SEM of three different samples. *denotes p < 0.05.

To determine the role of CFTR in DC, we compared gene expression in DC from CF and WT mice by microarray analysis. Nine genes were up-regulated in DC from CF mice compared to WT mice with more than 1.5- fold change in expression [see Additional file 1]. Interestingly, CFTR was expressed at 2.1-fold higher levels in DC from CF mice compared to WT mice. These higher levels of CFTR mRNA were also seen using real-time RT-PCR amplifying a fragment between exon 9 and 10, which is outside of the mutated region of CFTR gene in the CF mice, on independent samples (p < 0.05, Figure 2A). The absence of part of exon 10, the characteristic of the *Cftr*^tm1UNC ^mice genotype [40,41], was confirmed by RT-PCR (Figure 2B). This suggests increased levels of the mutant CFTR mRNA in the DC of the CF mice.

### Differentiation and Maturation of DC from WT and CF Mice

**Figure 3:**
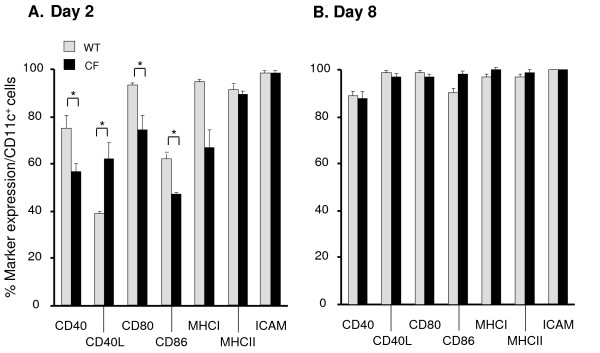
**Differentiation and maturation of DC from CF mice**. Differentiation and maturation of DC (CD11c^+^) from WT (gray) and CF (black) mice were monitored over time analyzing the surface expression of CD40, CD40L, CD80, CD86, MHCI, MHCII and ICAM. The y-axis represents the percentage expression of each marker in the CD11c population. Data from day 2 (**A**) and day 8 (**B**) are presented. Shown is the mean ± SEM of three different samples. *denotes p < 0.05.

In order to evaluate if the impaired CFTR expression in CF DC influences their differentiation profile, bone marrow cells were analyzed an day 0, 2, 4, 6 and 8 using the differentiation and maturation markers CD40, CD40L, CD80, CD86, ICAM, MHCI and MHCII. No quantitative or qualitative differences in the primary CD11c^+ ^bone marrow population between WT and CF mice were observed (data not shown). On day 2 there was a delay in the upregulation of CD40, CD80 and CD86 expression in the bone marrow culture of CF mice (p < 0.05, Figure 3A) whereas CD40L was increased in CF DC compared to the WT DC. On day 8, these differences were not observed anymore and the mature DC from the WT and CF mice expressed all markers comparably (Figure 3B).

### Downregulation of the Lipid Raft Protein Cav1 in DC from CF mice

**Figure 4:**
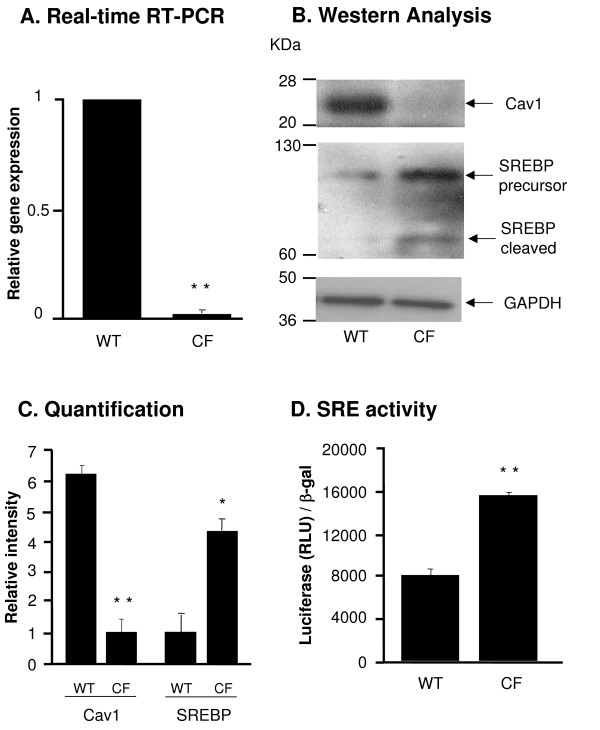
**Cav1 and SREBP expression in DC from WT and CF mice**. **A**. RNA was extracted from DC from WT and CF mice and Cav1 gene expression was measured by Real-time RT-PCR. Relative expression levels in the samples were calculated using the ΔΔCt method, with GAPDH as internal normalization control. The *y*-axis represents Cav1 cDNA transcription level in terms of relative quantity value (RQ). **B**. Western analysis of Cav1 and SREBP in DC from WT and CF mice and corresponding GAPDH expression. **C**. Quantification of Cav1 and SREBP expression by image intensity analysis normalized to GAPDH. **D**. Luciferase assay of SRE transcriptional activity in CF and WT DC. DC were infected with AdZ-SRE-luc for 48 h and harvested for luci-ferase assay and β-galactosidase assay. Data is shown luciferase activity (RLU) normalized to β-galactosidase. Shown is the mean ± SEM of three of independent samples. *denotes p < 0.05, ** denotes p < 0.01.

Seven genes were down-regulated in DC from CF mice with more than 1.5-fold change [see Additional file 2]. The expression level of the membrane lipid raft protein Cav1 in DC from the CF mice was 4.1-fold decreased compared to the WT mice. This finding was confirmed with real-time RT-PCR which showed a 50-fold reduction of the Cav1 mRNA level in the CF DC compared to WT DC (p < 0.01, Figure 4A). Cav1 protein was almost undetectable in CF DC (Figure 4B) and quantification of Cav1 protein expression level indicated a 6.2-fold lower expression in CF DC compared to WT DC (p < 0.01, Figure 4C). Cav1 is known to be negatively regulated by sterol regulatory element binding protein (SREBP) [30-32], therefore we further compared the expression and activity levels of SREBP in DC from CF and WT mice. SREBP functions as a transcription factor that binds and regulates the sterol regulatory element (SRE) containing promoter. The activation of SREBP requires the proteolytic cleavage to release the active form into nucleus and regulate the target genes [42]. The cleavage of SREBP protein was increased in the CF DC (Figure 4B) and quantification of the active form of SREBP demonstrated a 4.3-fold higher expression in DC of CF mice compared to WT mice (p < 0.05, Figure 4C). The transcriptional activity of SRE was increased in CF DC infected with AdZ-SRE-luc, an Ad vector expressing an SRE-promoter linked to a luciferase reporter, compared to WT controls infected with AdZ-SRE-luc (p < 0.01, Figure 4D), suggesting that SREBP activity was increased in the CF DC.

### Gene Expression Difference in DC from WT and CF Mice following *P. aeruginosa *Infection

To evaluate for differences in global gene expression between DC from WT and CF mice in response to *P. aeruginosa*, DC from WT and CF mice were infected with *P. aeruginosa *for 4 h, and gene expression profiles were evaluated by microarray analysis. Stimulation with *P. aeruginosa *induced changes in the expression of genes involved in inflammation and chemotaxis, signaling, cell cycling and apoptosis (Supplemental Tables 1 and 2). Especially, inflammation related genes were up-regulated upon the *P. aeruginosa *infection in both WT and CF mice, including 27 interferon-stimulated genes. Interestingly, 26 of 27 interferon-induced genes had higher fold changes of expression level in WT mice compared to the CF mice.

The expression levels of 30 lipid metabolism related genes were changed by more than 1.5-fold [see Additional file 3 and 4]. Among the genes with increased expression levels in WT and CF DC, Cav1 was upregulated 3.3-fold upon *P. aeruginosa *infection in WT mice (p < 0.05) and 2.6-fold in CF mice (p > 0.05). Among the genes which were downregulated upon *P. aeruginosa *infection (Supplemental Table 2), 7-dehydrocholesterol reductase (Dhcr7) was decreased 7.2-fold upon infection in WT mice (p < 0.05) but only 3.2-fold in CF mice (p > 0.05); the gene stearoyl-CoA desaturase 2 (Scd2) was downregulated 5.6-fold upon infection in WT mice (p < 0.05) but only 3.0-fold in CF mice (p > 0.05).

**Figure 5:**
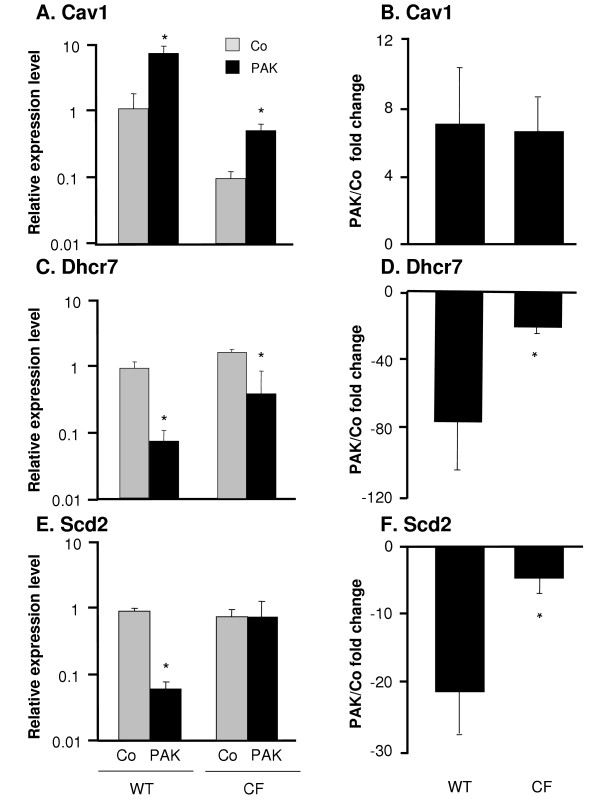
**Confirmation of microarray results by real-time RT-PCR**. DC from WT and CF mice were infected *in vitro *with *P. aeruginosa *for 4 h. RNA levels for three genes were measured by quantitative real-time RT-PCR. Relative expression levels in the samples were calculated using the ΔΔCt method, with GAPDH as internal normalization control. **A**, **C **and **E**. The *y*-axis represents the relative gene expression level for Cav1, Dhcr7 and Scd2 in the uninfected control DC (gray) and *P. aeruginosa *infected DC (black). **B, D, **and **F**. The *y*-axis represents fold change of Cav1, Dhcr7 and Scd2 expression upon *P. aeruginosa *infection compared to the control in both groups. Shown are the means ± SEM of three pairs of DC samples from WT and CF mice with or without *P. aeruginosa *infection. *denotes p < 0.05.

In order to confirm the microarray data, mRNA levels of these three genes were assessed by real-time RT-PCR of DC from independent experiments (Figure 5). Although basal expression level of Cav1 was lower in CF DC (p < 0.01) compared to WT DC, both groups responded to *P. aeruginosa *infection with a upregulation of Cav1 (p < 0.05, Figure 5A) resulting in similar fold change in the expression level after *P. aeruginosa *infection compared to the control (7.0-fold and 6.0-fold, Figure 5B). In contrast, the basal expression levels of Dhcr7 were comparable between CF and WT DC (Figure 5C) and decreased upon *P. aeruginosa *infection in both groups (p < 0.05). This resulted in 76-fold reduction upon exposure to *P. aeruginosa *in WT DC compared to 20-fold in CF DC leading to a difference in the fold change between two groups (p < 0.05, Figure 5D). The base line expression of Scd2 was also comparable between CF and WT DC, but only the WT DC showed a decreased response in Scd2 expression upon *P. aeruginosa *infection (p < 0.05, Figure 5E) resulting in a 21.2-fold decrease upon exposure to *P. aeruginosa *in WT DC compared to only 4.5-fold decrease in CF DC, elucidating a fold change difference of Scd2 expression between CF and WT mice (p < 0.05, Figure 5F).

**Figure 6:**
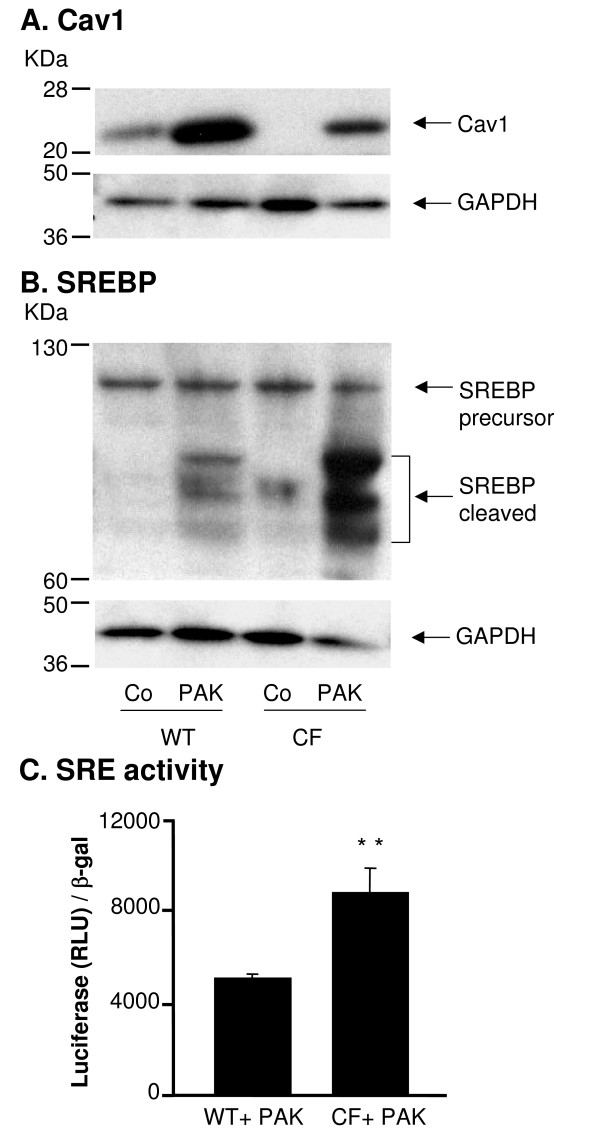
**Cav1 and SREBP expression in DC from WT and CF mice infected with *P. aeruginosa***. DC from WT and CF mice were infected *in vitro *with *P. aeruginosa *for 4 h, and uninfected cells served as the control (Co). A. Western analysis of Cav1 and corresponding GAPDH. B. Western analysis of SREBP and corresponding GAPDH. C. Luciferase assay of SRE transcriptional activity of CF and WT DC infected with *P. aeruginosa*. DC were infected with AdZ-SRE-luc for 48 h, and then infected with *P. aeruginosa *for 4 h. DC were harvested for luciferase assay and β-galactosidase assay. Data is shown luciferase activity (RLU) normalized to β-galactosidase. Shown is the mean ± SEM of three of independent samples. **denotes p < 0.01.

Further we addressed the question if the infection of *P. aeruginosa *in DC also leads to differences in the Cav1 and SREBP protein levels. As seen at the RNA level, Cav1 was upregulated in the presence of *P. aeruginosa *both in WT and CF DC, but expression levels were general higher in the WT mice (Figure 6A). The same tendency was observed in the SREBP protein level, but with a higher baseline expression level in the CF mice (Figure 6B). The transcriptional activity of SRE was also higher in CF DC followed *P. aeruginosa *infection than the WT controls (p < 0.01, Figure 6C). These data suggested a strong correlation between the presence of CFTR and expression of lipid metabolism related genes that are differently expressed in the CF DC in response to the *P. aeruginosa *infection compared to the WT DC.

## Discussion

Lung disease in CF is characterized by an exaggerated inflammatory state and chronic infection with *P. aeruginosa *[38]. As the responses of the immune system are not adequate to eradicate *P. aeruginosa *from the lung in CF, the present study evaluates the general role of CFTR in DC, the most critical antigen presenting cells in initiating and regulating antigen specific immune responses [26-29].

CFTR was expressed in DC, and bone marrow cells from CF mice showed a delay in the differentiation into DC compared to the WT mice. DC derived from CF mice showed relatively few differences in basal gene expression compared to WT DC, including a lipid raft gene Cav1 with lower expression in CF DC. Consistently, expression and activity of the sterol regulatory element binding protein (SREBP), a negative regulator of Cav1 expression, was increased in CF DC. Following infection with *P. aeruginosa *gene expression between CF and WT DC differed for a number of genes. Of these, Dhcr7 and Scd2, two members of the lipid metabolism enzymes that are also regulated by SREBP, were found to be differently regulated.

### CFTR in DC

Expression of CFTR in DC has so far not been reported. DC play an important part in antigen presentation and stimulation of T cells and are present in the lung in a network [26,27,43]. The CFTR expression levels in the DC were lower compared to the whole lung.

The levels of the non-mutated part of CFTR mRNA were increased in the CF DC. This is in contrast to previous studies using the same microarray chip on RNA from lung, pancreas and small intestine tissue of CF mice with the identical CFTR mutation (*Cftr*^tm1UNC^) [44-48]. The *Cftr*^tm1UNC ^mouse has an insertion of a premature termination condon into exon 10 of CFTR gene [40,41], and this mutation has been reported to activate an alternative splicing and result in a in-frame deletion, indicating that the cells may produce a CFTR protein with impaired function [49]. The murine CFTR transcripts were detected in tracheal tissue from *Cftr*^tm1UNC ^mouse with similar level compared to the WT mice [50], and the presence of CFTR protein was also reported in mesenchymal connective tissue from *Cftr*^tm1UNC ^mice [51]. This could suggest that the regulation of CFTR mRNA expression in *Cftr*^tm1UNC ^may vary in different tissue or cell types. The increased mRNA levels of CFTR in CF DC could be due to increased transcription or stability of the mRNA in the DC background.

### Differentiation of Bone Marrow-derived DC from CF Mice

Bone marrow cells from CF mice showed a delay in the early phase of differentiation into DC compared to the WT mice, with lower expression of co-stimulatory molecules. Maturation and differentiation of DC are crucial in initiation and regulation of immune response, such as T cells activation and cytokine secretion [26-28]. In CF infants, CFTR mutation itself could produce an inflammatory milieu in the airway even in absence of pathogen infection, suggesting dysfunctional immune regulation [19]. Slowed differentiation of DC could lead to reduced inhibitory regulation of inflammatory mediators, and it could be direct effect of deficient CFTR expression. Perez *et al *created a CF cell model by using CFTR specific inhibitor CFTRinh-172 in normal bronchial epithelial cells, indicating that CFTR inhibition alone is sufficient to produce an exaggerated inflammatory response [52].

### Basal Gene Expression Differences in CFTR-deficient DC

Few changes in basal gene expression were seen comparing DC derived from CF and WT mice. The previous studies analyzing gene expression in tissues affected by CF in mice, including lung, pancreas and small intestine, found different expression for a larger number of genes [44-48]. As the RNA in these studies was derived from tissues containing a variety of cell types, a direct comparison with the results of the present study is difficult. It is possible that cultured cells respond differently compared to the cells *in vivo*, as the DC are cultured in enhanced medium with supplemented cytokines. However our gene profile study could still provide an insight in the influence of CFTR on function of DC.

Cav1 mRNA and protein were decreased in the CF DC compared to the WT DC. Caveolin is the principal component of caveolae, lipid domains characterized by a flask-shaped invaginated morphology, which play a role in endocytosis, signal transuction and the cellular transport of cholesterol [53,54]. Cav1 has not been previously reported to be directly affected in CF. However, CFTR was found to localize to lipid rafts membrane fractions characterized by an enrichment of Cav1 [25]. The co-localization of CFTR and Cav1 could suggest a potential interaction between the two proteins. Cav1 expression is negatively regulated by SREBP, a critical factor in cellular lipid [30-32]. Activation of SREBP, using a promoter reporter assay, has been reported in CF cells [55]. The elevated expression and activity of SREBP could be the underlying factor for the decreased Cav1 expression.

### *P. aeruginoasa *Induced Gene Expression Changes in CF DC

Changes in the expression level of 912 genes were induced by *P. aeruginosa *infection. Most genes belonged to the functional categories of inflammation, signaling, metabolism and transcription *etc*. The majority of up-regulated genes were immune response related genes (112 of 465), which presents the typical character of DC upon pathogen infection. A multiple correction is one strategey to confront the problem of false positives in microarray study. However our study and other similar studies cannot count with sample size sufficiently large to afford a multiple test comparison. As physiological effect are often small in magnitude and rather than missing potentially important observation, we chose to forgo the use of multiple comparison in favor of confirmation by an independent method (TaqMan RT-PCR), of those observations that are most biologically relevant for our study system. The results that are not confirmed by RT-PCR could be tentative.

The magnitudes of gene expression changes were mostly larger in WT mice than CF mice (782 of 912); especially 27 interferon/interleukin induced genes. This suggests that defective CFTR may affect the proper immune response of DC against the *P. aeruginosa *infection. This observation is in agreement with the fact that the presence of WT CFTR in human bronchial epithelial cell positively influenced cytokines of innate immunity in response to *P. aeruginosa *such as interleukin-8 (IL-8), IL-6, CXCL1, indicating CFTR plays a role in resistance to *P. aeruginosa *[56].

The expression levels of 30 lipid metabolism related genes were changed by more than 1.5-fold (13 up-regulated genes and 17 down-regulated genes). Cav1, which was virtually absent in non-infected CF DC, was increased upon the *P. aeruginosa *infection with similar fold change in WT and CF mice. The LPS stimulation in endothelial cells induces the expression of Cav1 in a NF-κB-dependent manner [57]. It might serve as an underlying mechanism of upregulation of Cav1 expression in DC followed with *P. aeruginosa *infection.

In contrast, two other lipid metabolism related genes, Dhcr7 and Scd2, were strongly decreased in WT DC, but only to a much lesser extent in CF DC. Dhcr7 converts dehydrocholesterol (DHC) to cholesterol, and Dhcr7 deficiency in human leads to a syndrome characterized by immunological changes [58,59]. Stearoyl-CoA desaturase (SCD) is an enzyme that catalyzes the Δ9-cis desaturation of saturated fatty acyl-CoA [60]. Both Dhcr7 and Scd2 genes contain a sterol-regulatory element, the binding site for the transcription factor SREBP, in their promoter regions. In contrast to Cav1, Dhcr7 and Scd2 expressions are up-regulated by the active form of SREBP [36,37]. *P. aeruginosa *infection induces the apoptosis of the host cells [61], and SREBP is cleaved during programmed cell death [62]. Sphingolipid storage caused by the haemolytic phospholipase C of *P. aeruginosa *stimulated the SREBP-1 activation [63], and induced accumulation of intracellular cholesterol [64]. As elevated expression and activity of SREBP were present in CF DC after *P. aeruginosa *infection compared to WT DC, it may lead to a compensatory upregulation of Dhcr7 and Scd2 that results in a more moderate reduction of these genes.

The present study indicates that, even if expressing at a low level in immune cells such as DC, CFTR influences cellular lipid metabolism, possibly through increased levels of active SREBP. It has been shown that the fatty acid abnormalities in CFTR-deficient tissues positively correlate with chronic or acute inflammation, suggesting the important role of lipid homeostasis in the regulation of the innate host immune response [16]. The defective CFTR expression in DC may affect lipid raft composition, pathogen uptake and clearance, intracellular signaling events, and give rise to inadequate inflammatory responses.

## Abbreviations

CF: cystic fibrosis; CFTR: cystic fibrosis transmembrane conductance regulator; DC: dendritic cells; CF mice: CFTR knockout mice; WT mice: wild type mice; SREBP: sterol regulatory element binding protein; SRE: sterol regulatory element; Dhcr7: 3β-hydroxysterol-Δ7 reductase; Scd2: stearoyl-CoA desaturase 2.

## Competing interests

The authors declare that they have no competing interests.

## Authors' contributions

YX carried out part of the experiments, analyzed the data and wrote the draft of the manuscript. CT carried out part of the experiments and the microarray analysis. AK participated in the flow cytometory analysis. LQ participated design and analysis of part of the experiment. RC participated in the design of the study. SW conceived of the study, and participated in its design and coordination and helped to draft the manuscript. All authors read and approved the final manuscript.

## Supplementary Material

Additional file 1 **Up-regulated Genes in DC from CF Mice Compared to WT Mice**. The data provided a table of genes up-regulated in DC from CF mice compared to WT mice. Click here for file

Additional file 2 **Down-regulated Genes in DC from CF Mice Compared to WT Mice**. the data provided a table of genes down-regulated in DC from CF mice compared to WT mice. Click here for file

Additional file 3 **Up-regulated Lipid Metabolism-related Genes in DC from WT and/or CF Mice following *P. aeruginosa *Infection**. The data provided a table of lipid metabolism-related genes up-regulated in DC from WT and/or CF mice following *P. aeruginosa *infection. Click here for file

Additional file 4 **Down-regulated Lipid Metabolism-related Genes in DC from WT and/or CF Mice following *P. aeruginosa *Infection**. The data provided a table of lipid metabolism-related genes down-regulated in DC from WT and/or CF mice following *P. aeruginosa *infection. Click here for file
