# Supplementary material for: Influence of the cystic fibrosis transmembrane conductance regulator on expression of lipid metabolism-related genes in dendritic cells
Source: Respir Res. 2009 Apr 3;10(1):26. doi: 10.1186/1465-9921-10-26 (PMC2683168; doi:10.1186/1465-9921-10-26)
Supplement: Additional file 1 — Up-regulated Genes in DC from CF Mice Compared to WT Mice. The data provided a table of genes up-regulated in DC from CF mice compared to WT mice. [file 1465-9921-10-26-S1.pdf]

Table 1. Up-regulated Genes in DC from CF Mice Compared to WT Mice

Category / Probe Set ID	Gene Symbol	Gene Title	CF-Co / WT-Co [(a)]	p value[(b)]
**signal transduction / growth control**				
161181_f_at	Dusp16	dual specificity phosphatase 16	4.42	0.045
99915_at	Areg	amphiregulin	3.44	0.005
102698_at	Epas1	endothelial PAS domain protein 1	1.73	0.005
**metabolism / enzyme**				
100016_at	Mmp11	matrix metallopeptidase 11	2.29	0.014
**transport protein**				
94758_s_at	Cftr	cystic fibrosis transmembrane conductance regulator homolog	2.14	0.001
101625_at	Kcns2	K+ voltage-gated channel, subfamily S, 2	1.82	0.016
100982_at	Kctd9	potassium channel tetramerisation domain containing 9	1.51	0.017
**secreted protein**				
93956_at	Ifit3	interferon-induced protein with tetratricopeptide repeats 3	5.06	0.026
104447_r_at	4933428G09Rik	RIKEN cDNA 4933428G09 gene	1.60	0.011

(a) Geometric mean ratio of gene expression levels in DC from CF mice vs. WT mice; n = 6 per-group.
(b) P value based on comparison of gene expression levels in DC of CF mice with WT mice.
