# Supplementary material for: Influence of the cystic fibrosis transmembrane conductance regulator on expression of lipid metabolism-related genes in dendritic cells
Source: Respir Res. 2009 Apr 3;10(1):26. doi: 10.1186/1465-9921-10-26 (PMC2683168; doi:10.1186/1465-9921-10-26)
Supplement: Additional file 2 — Down-regulated Genes in DC from CF Mice Compared to WT Mice. the data provided a table of genes down-regulated in DC from CF mice compared to WT mice. [file 1465-9921-10-26-S2.pdf]

Table 2. Down-regulated Genes in DC from CF Mice Compared to WT Mice

Category / Probe Set ID	Gene Symbol	Gene Title	CF-Co / WT-Co [(a)]	p value[(b)]
**signal transduction / growth control**				
160280_at	Cav1	caveolin, caveolae protein 1	-4.14	0.006
97549_at	Cfl2	cofilin 2, muscle	-1.55	0.010
95546_g_at	Igf1	insulin-like growth factor 1	-1.51	0.047
**metabolism / enzyme**				
93235_at	BB128963	expressed sequence BB128963	-1.92	0.007
93299_at	Msh6 / LOC633695	mutS homolog 6 (E. coli) similar to mutS homolog 6	-1.82	0.048
92978_s_at	Serpinb2	serine (or cysteine) peptidase inhibitor, clade B, member 2	-1.75	0.034
97227_at	Gna12	guanine nucleotide binding protein, alpha 12	-1.60	0.049

(a) Geometric mean ratio of gene expression levels in DC from CF mice vs. WT mice; n = 6 per-group.
(b) P value based on comparison of gene expression levels in DC of CF mice with WT mice.
