# Supplementary material for: Influence of the cystic fibrosis transmembrane conductance regulator on expression of lipid metabolism-related genes in dendritic cells
Source: Respir Res. 2009 Apr 3;10(1):26. doi: 10.1186/1465-9921-10-26 (PMC2683168; doi:10.1186/1465-9921-10-26)
Supplement: Additional file 3 — Up-regulated Lipid Metabolism-related Genes in DC from WT and/or CF Mice following P. aeruginosa Infection. The data provided a table of lipid metabolism-related genes up-regulated in DC from WT and/or CF mice following P. aeruginosa infection. [file 1465-9921-10-26-S3.pdf]

Table 3. Up-regulated Lipid Metabolism-related Genes in DC from WT and/or CF Mice following *P.aeruginosa* Infection

Probe Set ID	Gene Symbol	Gene Title	n-fold up-regulation [(a)] PAK / Co WT	p value[(b)]	PAK / Co CF	p value[(b)]
104647_at	Ptgs2	prostaglandin-endoperoxide synthase 2	131.93	0.000	118.30	0.005
104406_at	Ptges	prostaglandin E synthase	7.58	0.000	8.96	0.013
94214_at	Fabp3	fatty acid binding protein 3, muscle and heart	6.91	0.016	5.37	0.057
96662_at	Ppap2b	phosphatidic acid phosphatase type 2B	5.85	0.003	4.78	0.060
104509_at	Ch25h	cholesterol 25-hydroxylase	5.54	0.008	4.34	0.087
94507_at	Acsl1	acyl-CoA synthetase long-chain family member 1	3.48	0.000	3.37	0.030
160280_at	Cav1	caveolin, caveolae protein 1	3.28	0.017	2.61	0.384
99592_f_at	Rdh11	retinol dehydrogenase 11	2.66	0.001	2.07	0.038
160171_f_at	Acot9	acyl-CoA thioesterase 9	1.75	0.017	1.32	0.379
104017_at	Acsl4	acyl-CoA synthetase long-chain family member 4	1.73	0.027	1.62	0.168
99513_at	Pla2g4a	phospholipase A2, group IVA (cytosolic, calcium-dependent)	1.70	0.011	1.39	0.466
102370_at	Hsd17b11	hydroxysteroid (17-beta) dehydrogenase 11	1.64	0.006	1.40	0.030
102659_at	Cln3	ceroid lipofuscinosis, neuronal 3, juvenile (Batten, Spielmeyer-Vogt disease)	1.62	0.021	1.63	0.195

(a) Geometric mean ratio of gene expression levels of *P. aeruginosa* infected DC samples/uninfected DC samples; n = 6 per condition
(b) P value based on comparison of *P. aeruginosa* infected DC with uninfected controls
