# Supplementary material for: Influence of the cystic fibrosis transmembrane conductance regulator on expression of lipid metabolism-related genes in dendritic cells
Source: Respir Res. 2009 Apr 3;10(1):26. doi: 10.1186/1465-9921-10-26 (PMC2683168; doi:10.1186/1465-9921-10-26)
Supplement: Additional file 4 — Down-regulated Lipid Metabolism-related Genes in DC from WT and/or CF Mice following P. aeruginosa Infection. The data provided a table of lipid metabolism-related genes down-regulated in DC from WT and/or CF mice following P. aeruginosa infection. [file 1465-9921-10-26-S4.pdf]

Table 4. Down-regulated Lipid Metabolism-related Genes in DC from WT and/or CF Mice following *P.aeruginosa* Infection

Probe Set ID	Gene Symbol	Gene Title	n-fold up-regulation [a] PAK / Co WT	p value[b]	PAK / Co CF	p value[b]
98989_at	Dhcr7	7-dehydrocholesterol reductase	-7.19	0.019	-3.20	0.080
95758_at	Scd2	stearoyl-Coenzyme A desaturase 2	-5.58	0.001	-3.04	0.168
95597_at	Ptgs1	prostaglandin-endoperoxide synthase 1	-4.93	0.006	-2.95	0.082
93320_at	Cpt1a	carnitine palmitoyltransferase 1a, liver	-3.48	0.007	-2.63	0.072
101426_at	Cerk	ceramide kinase	-2.51	0.000	-2.21	0.163
103299_at	Pld4	phospholipase D family, member 4	-2.40	0.002	-2.20	0.195
100066_at	Gart	phosphoribosylglycinamide formyltransferase	-2.05	0.006	-1.75	0.146
94056_at	Scd1	stearoyl-Coenzyme A desaturase 1	-1.94	0.024	-1.53	0.250
92474_at	Pld1	phospholipase D1	-1.93	0.029	-1.71	0.609
104003_at	Phka2	phosphorylase kinase alpha 2	-1.82	0.009	-2.11	0.201
100099_at	Smpd1	sphingomyelin phosphodiesterase 1, acid lysosomal	-1.75	0.025	-1.68	0.219
103401_at	Acads	acyl-Coenzyme A dehydrogenase, short chain	-1.74	0.014	-1.32	0.453
160770_at	Mvd	mevalonate (diphospho) decarboxylase	-1.67	0.007	-1.47	0.256
98909_at	Lias	lipoic acid synthetase	-1.66	0.036	-1.44	0.301
94872_at	Smpdl3a	sphingomyelin phosphodiesterase, acid-like 3A	-1.66	0.031	-1.51	0.353
161074_at	Pcyt1a	phosphate cytidylyltransferase 1, choline, alpha isoform	-1.55	0.045	-1.57	0.207
97515_at	Hsd17b4	hydroxysteroid (17-beta) dehydrogenase 4	-1.50	0.040	-1.50	0.369

(a) Geometric mean ratio of gene expression levels of *P. aeruginosa* infected DC samples/uninfected DC samples; n = 6 per condition
(b) P value based on comparison of *P. aeruginosa* infected DC with uninfected controls
